# Draft genome sequence of *Enterococcus* sp. SB12 isolated from artisanal cheese of the Carpathian

**DOI:** 10.1128/MRA.00865-23

**Published:** 2023-11-29

**Authors:** Viktoriia Mushynska, Ivan Roman, Stepan Tistechok, Iryna Slyvka, Orysia Tsisaryk, Oleksandr Gromyko, Oksana Shtapenko, Vasyl Syrvatka

**Affiliations:** 1 Laboratory of Reproductive Biotechnology, Institute of Animal Biology National Academy of Agrarian Sciences of Ukraine, Lviv, Ukraine; 2 Genetic and Biotechnology Department, Ivan Franko National University of Lviv, Lviv, Ukraine; 3 Stepan Gzhytskyi National University of Veterinary Medicine and Biotechnologies of Lviv, Lviv, Ukraine; Rochester Institute of Technology, Rochester, New York, USA

**Keywords:** *Enterococcus*, artisanal cheese, genomic DNA

## Abstract

In this study, we present the draft genome sequence of the *Enterococcus* sp. strain SB12, which was isolated from artisanal cheese of the Carpathian region (Ukraine). The *de novo* assembly produced 64 contigs, with a total length of 2,514,601 bp. Phylogenetic analysis revealed its proximity to the *Enterococcus faecium* strains.

## ANNOUNCEMENT

Enterococci belong to the lactic acid bacteria group; they are common residents of mammals’ gastrointestinal tracts and are characterized by their various distinctive properties that are especially useful in the biotechnology industry. Today, only some *Enterococcus faecium* strains are permitted for use in the food industry, prior to which they must be differentiated from pathogenic strains, as determined by the European Food Safety Authority (1, 2). To avoid all possible risks for consumers, genome sequencing analysis of *Enterococcus* strains can help distinguish between safe and potentially harmful strains (3, 4).

The *Enterococcus* sp. SB12 strain was isolated from a traditional cheese called “brynza” by direct inoculation of a homogenate of cheese samples in sterile saline on De Man-Rogosa-Sharp medium (BTL, Poland) and incubated at 45°C for 48–72 hours (5). For the total DNA extraction, the *Enterococcus* sp. SB12 strain was inoculated into 20 mL Tryptic Soy Broth medium and incubated at 37°C for 12 hours. Genomic DNA was extracted using a DNeasy PowerLyzer Microbial Kit (Qiagen, Valencia, CA, USA). The concentration and quality of the extracted DNA were assessed using the A260/280 ratio on a DS-11 spectrophotometer (DeNovix Inc., USA), and electrophoresis was performed in 1% agarose gel. The obtained DNA was sequenced by Explogen LLC (Lviv, Ukraine) using an Illumina paired-end sequencing library (TruSeq Sample Preparation Kit, Illumina, USA), as recommended by the manufacturer. The 2 × 250 bp paired-end read sequencing was performed on an Illumina MiniSeq instrument (Illumina, Inc.). Read quality was controlled using the FASTQC quality control tool version 0.11.9 using default settings.

The Illumina sequencing data were *de novo* assembled using Velvet Assembler version 1.2.10 using default settings (6). The assembly yielded 64 contigs, with a total length of 2,514,601 bp, an *N*_50_ value of 58,793 bp, and an *L*_50_ value of 14. This strain had a GC content of 38.1% and an average coverage of 424.0×. Annotation using NCBI Prokaryotic Genome Annotation system version 6.6 (7) revealed that the strain harbored 2,339 protein-coding sequences (Table 1).

**TABLE 1:** Genomic features of *Enterococcus* sp. SB12 isolated from “brynza” cheese

Parameter	Value
Total length (bp)	2,514,601
No. of contigs	64
GC content (%)	38.1
Size of the longest scaffold (bp)	143,397
*N*_50_ (bp)	58,793
*L*_50_	14
No. of protein-coding genes	2,339
No. of rRNAs	4
No. of tRNAs	55
Estimated completeness (%)	98.07

**Fig 1:**
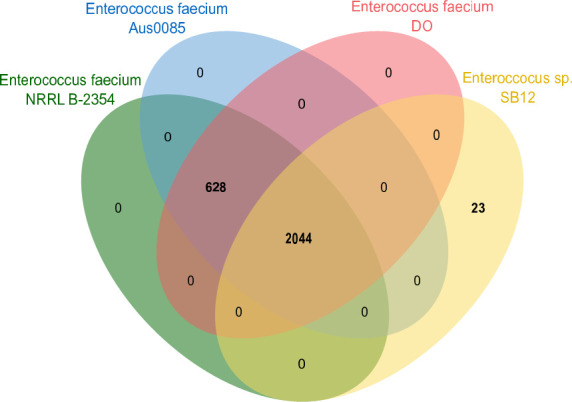
Venn diagram of the orthologous gene clusters present among *Enterococcus* sp. SB12 and the closest taxonomically defined species of the *Enterococcus* genus. Cluster and protein counts indicate the number of categories in the functions of the orthologous genes and the total number of orthologous genes in each category, respectively.

Phylogenetic analysis using the Automated Multi-Locus Species Tree (autoMLST) (8) server showed that the closest taxonomically defined species were *E. faecium* NRRL B-2354 (GenBank accession number CP004063), *E. faecium* Aus0085 (GenBank accession number CP006620), and *E. faecium* DO (GenBank accession number CP003583). To examine the differences between these strains, the compositions of orthologous gene clusters were analyzed using OrthoVenn3 (Fig. 1) (9). In total, 2,044 clusters were found to be common among the four *Enterococcus* strains. At the same time, 628 clusters were common to the strains NRRL B-2354, Aus0085, and DO that were not observed in the SB12 strain. In turn, the SB12 strain had 23 unique clusters involved in the processes of biosynthesis and catabolism of compounds, transmembrane transport, as well as clusters with unknown function.

The findings suggest that SB12 is probably a novel strain of *Enterococcus faecium*, but further research is needed to confirm this. Accordingly, this is the first report of a draft genome sequencing of Enterococci isolated from “brynza” cheese.

## Data Availability

This study project is available under NCBI BioProject accession number PRJNA1006558 and BioSample accession number SAMN37043343. The assembly and the reads used to assemble the genome are available under GenBank accession number JAVFKT000000000 and Sequence Read Archive (SRA) accession number SRR25731394, respectively.
